# Development of DNA sensor crustacean allergen analysis in food products

**DOI:** 10.1186/2045-7022-1-S1-P14

**Published:** 2011-08-12

**Authors:** Nisamanee Charoenchon, Piyasak Chaumpluk

**Affiliations:** 1 Chulalongkorn University, Department of Botany, Faculty of Science, Bangkok, Thailand

## 

Crustaceans is one of the most common causes of food allergies. Its persistency is life-threatening therefore, method to detect the presence of crustacean constituent in food products would be indispensable to ensure the safety. We have developed a simple and rapid method to detect the presence of crustacean residues in food products through the allergenic *Pen* gene. Detection method was based on isothermal DNA amplification using primers specific to target *Pen* genes. Detection of DNA products was based on fluorescence visualization on UV light source after competitive DNA hybridization using fluorescence beta-pyrroridinyl peptide nucleic acid probe and its corresponding quencher. This method was crustacean specific and could detect shrimp, prawn, crab and lobster residues. Its sensitivity was sufficient to detect 20 copies of specific *Pen* DNA to *Pen* gene. Presence of shrimp, prawn, crab and lobster constituents were able to detect within 2 hours at DNA level without relying on laboratory facilities. This method should provide a benefit to food manufacturers, clinical doctors and allergenic patients by providing rapid information on the presence of crustacean contamination in foods.

